# Sonoclot Signature Analysis in Patients with Liver Disease and Its Correlation with Conventional Coagulation Studies

**DOI:** 10.1155/2013/237351

**Published:** 2013-12-11

**Authors:** Priyanka Saxena, Chhagan Bihari, Archana Rastogi, Savita Agarwal, Lovkesh Anand, Shiv Kumar Sarin

**Affiliations:** ^1^Department of Hematology, Institute of Liver and Biliary Sciences, D-1, Vasant Kunj, New Delhi 110070, India; ^2^Department of Pathology, Institute of Liver and Biliary Sciences, D-1, Vasant Kunj, New Delhi 110070, India; ^3^Department of Hepatology, Institute of Liver and Biliary Sciences, D-1, Vasant Kunj, New Delhi 110070, India

## Abstract

*Introduction*. Liver disease patients have complex hemostatic defects leading to a delicate, unstable balance between bleeding and thrombosis. Conventional tests such as PT and APTT are unable to depict these defects completely. *Aims*. This study aimed at analyzing the abnormal effects of liver disease on sonoclot signature by using sonoclot analyzer (which depicts the entire hemostatic pathway) and assessing the correlations between sonoclot variables and conventional coagulation tests. *Material and Methods*. Clinical and laboratory data from fifty inpatients of four subgroups of liver disease, including decompensated cirrhosis, chronic hepatitis, cirrhosis with HCC and acute-on-chronic liver failure were analyzed. All patients and controls were subjected to sonoclot analysis and correlated with routine coagulation parameters including platelet count, PT, APTT, fibrinogen, and D-dimer. *Results*. The sonoclot signatures demonstrated statistically significant abnormalities in patients with liver disease as compared to healthy controls. PT and APTT correlated positively with SONACT (*P* < 0.008 and <0.0015, resp.) while platelet count and fibrinogen levels depicted significant positive and negative correlations with clot rate and SONACT respectively. *Conclusion*. Sonoclot analysis may prove to be an efficient tool to assess coagulopathies in liver disease patients. Clot rate could emerge as a potential predictor of hypercoagulability in these patients.

## 1. Introduction

Patients with liver disease show significant changes in the hemostatic system. Consequently, routine diagnostic tests such as platelet count, prothrombin time (PT), and activated partial thromboplastin time (APTT) are frequently abnormal. However, interpretation of these tests is much less accurate in patients with complex hemostatic disorders as can be found in patients with liver disease [1]. It is now established that patients with liver disease not only have bleeding tendencies but may develop thrombotic complications as well [2].

The inability of PT-INR and APTT to predict the bleeding risk can be explained by the fact that they incompletely reflect the coagulation process. The parallel decline in the level of natural anticoagulants leading to a prothrombotic tendency is not depicted by these tests. Additionally, significant variations in the INR values have been reported in liver disease patients when tested in different laboratories. Due to this poor reproducibility of INR values, models for end stage liver disease (MELD) score variations up to 12 points have been noted [3]. This could lead to significant discrepancies in the management of these patients.

Standard coagulation tests such as PT and APTT do not incorporate cellular elements. They tend to provide data on isolated aspects of coagulation cascade and overlook factors such as rate of clot formation, time taken for maximal clot retraction, and maximal clot strength. Instead, viscoelastic devices such as sonoclot provide *in vitro* assessment of global coagulation. Sonoclot may also be useful in diagnosing systemic fibrinolysis, though it may not reflect localized clot breakdown by plasmin. Most conventional coagulation tests end when the first fibrin strands are developing, whereas viscoelastic coagulation tests begin at this point and continue throughout clot development, retraction, and lysis [4].

This study was carried out to analyze the abnormalities of sonoclot signature in patients with liver diseases including chronic hepatitis, decompensated cirrhosis, compensated cirrhosis with hepatocellular carcinoma, and acute-on-chronic liver failure. The sonoclot signature parameters studied included sonoclot activated clotting time (SONACT), clot rate (CR), platelet function (PF), time to peak (TP), peak amplitude (PA), and R2 peak character. We also aimed to establish a correlation between the above mentioned sonoclot parameters and conventional coagulation tests like PT, international normalized ratio (INR), APTT, fibrinogen levels, platelet count, and D-dimer levels in these patients.

## 2. Sonoclot Coagulation and Platelet Function Analyzer, Sienco Inc., Arvada, CO, USA

The sonoclot analyser was introduced by von Kaulla et al. in 1975. Sonoclot measurements are based on detection of viscoelastic changes in the whole blood sample. The instrument provides information on the entire hemostatic process in the form of a qualitative graph known as sonoclot signature along with several quantitative measurements [5].

**Figure 1:**
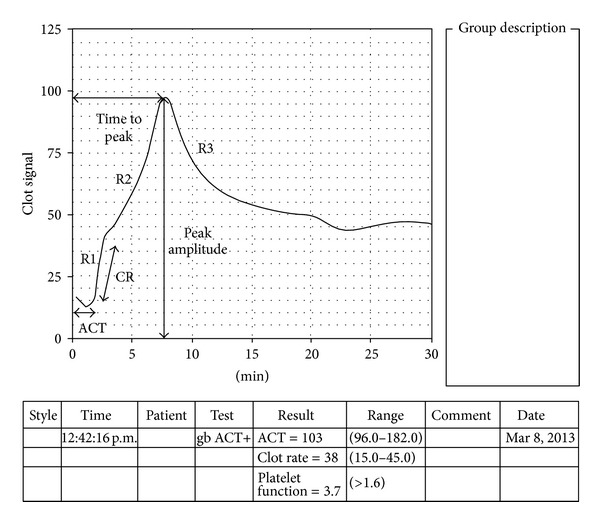
Normal sonoclot signature ACT (SONACT: activated clotting time), CR: clot rate.

The quantitative measurements include sonoclot activated clotting time (SONACT) which is the onset time in seconds till the beginning of fibrin formation. The rate of fibrin formation from fibrinogen is depicted by the gradient of primary slope (R1) and is known as clot rate (CR). It is expressed in units per minute. The secondary slope (R2) reflects fibrin polymerization and platelet-fibrin interaction. The R2 peak indicates completion of fibrin formation and has two variables, the time to peak (in minutes), which is an index of the rate of conversion of fibrinogen to fibrin, and peak amplitude (expressed in units), which is an index of fibrinogen concentration. The downward slope (R3) after the peak is produced as platelets induce contraction of the completed clot. In cases of low platelet counts and/or poor platelet function, a shallow R3 slope is obtained. Hence, the R3 slope gradient determines the number of available platelets and the level of platelet function and is recorded as platelet function (PF) by the analyzer (Figure 1). In patients with accelerated fibrinolysis, the decrease in signal after the R3 slope can be clinically used as a measure of fibrinolysis [6].

## 3. Material and Methods

### 3.1. Patients and Control

An observational study was carried out over a period of three months wherein data of 50 adult inpatients without any anticoagulation therapy with liver disease in a superspeciality liver institute were analyzed. The study also included 10 healthy controls from voluntary donors at the blood bank. None of the candidates in the control group had any other apparently known disease. Exclusion criteria for controls were presence of any chronic medical condition (especially coagulopathies), patients on anticoagulation, and individuals on long term medications.

Patients were classified into four groups:

1. Group 1 (G1) or decompensated cirrhosis (D. cirrhosis) included 16 (32%) patients with decompensated cirrhosis which was defined by histological presence of regenerative nodules surrounded by fibrosis with clinical stage 3 or 4 [7] along with presence of ascites, variceal haemorrhage, encephalopathy, or jaundice [8]. This group comprised eight patients with alcoholic cirrhosis, one patient with hepatitis C-related cirrhosis, five patients with cryptogenic cirrhosis, and two patients with nonalcoholic steatohepatitis (NASH) induced cirrhosis.
2. Group 2 (G2) or chronic hepatitis (CH) included 14 (28%) patients with chronic liver disease (CLD) other than cirrhosis (chronic hepatitis group). This group comprised four patients with chronic alcoholic hepatitis, seven patients with chronic viral hepatitis, and three patients with chronic cholestatic hepatitis.
3. Group 3 (G3) or cirrhosis included 11 (22%) patients with compensated cirrhosis who had an additional finding of hepatocellular carcinoma (HCC).
4. Group 4 (G4) or acute-on-chronic liver failure (ACLF) included nine (18%) patients with acute on chronic liver failure (ACLF) as defined by the Asia Pacific Association for the Study of the Liver (APASL). The APASL's definition of ACLF is “acute hepatic insult manifesting as jaundice and coagulopathy, complicated within 4 weeks by ascites and/or encephalopathy in a patient with previously diagnosed or undiagnosed chronic liver disease” [9].
5. A fifth group (control group) was created which comprised 10 voluntary healthy controls.

Patients in G3 (cirrhosis) were segregated from G1 (D. cirrhosis) (though both groups consisted of patients with cirrhosis) because there is sufficient recent evidence to indicate that compensated and decompensated cirrhosis are two separate entities and should be analyzed separately [8, 10]. In our study, the patient group of compensated cirrhosis had an additional finding of HCC.

### 3.2. Demographic Data and Clinical Presentation

The clinical profile of the patients including age, sex, clinical presentation, underlying liver disease, and bleeding history was recorded and summarized in Table 1.

There were 34 male (68%) and 16 female (32%) patients. The minimum age was 29 years while the maximum age was 70 years with a mean age of 50.7 years (SD ± 10.68). The control group consisted of 6 male (60%) and 4 female (40%) patients. The minimum age was 20 years and the maximum was 52 years with a mean age of 34.2 years (SD ± 9.4).

The most common cause in G1 (D. cirrhosis) was alcoholic liver disease. Eight patients (50%) in the group were clinically reported to have hepatic encephalopathy. Additionally, four patients (44.4%) in G4 (ACLF) also had hepatic encephalopathy. A total of 13 patients out of 50 (26%) developed features of sepsis, six of which belonged to G1 (D. cirrhosis) (37.5%), three belonged to G2 (CH) (21%), one belonged to G3 (cirrhosis) (9%), and three belonged to G4 (ACLF) (33%). History of bleeding was present in six patients (12%) who included four patients from G1 (D. cirrhosis), one patient from G2 (CH), and one patient from G3 (cirrhosis) (Table 1). Bleeding from varices was the commonest with four patients (66.7%) having history of variceal bleed (multiple emesis and/or melena), one patient with history of intra-abdominal bleed, and one patient having mucosal bleeds.

### 3.3. Laboratory Tests

The platelet count of all the patients as well as control group was carried out on a hematology autoanalyzer (Coulter Hmx Hematology Analyser; Beckman Coulter Inc., Brea, California, USA).

For coagulation parameters, blood from patients and controls was collected in two citrated tubes containing buffered sodium citrate (0.109 M, 3.2%) in the ratio blood : anticoagulant 9 : 1. The citrated samples were processed within half an hour of collection. One of the tubes containing citrated blood was centrifuged and plasma was obtained. The plasma was run on fully automated coagulometer (Sysmex CA 1500; Sysmex Corporation, Kobe, Japan) and values of PT, INR, APTT, and fibrinogen were recorded. The remainder of the plasma was used for determination of D-dimer levels by a semiquantitative rapid latex agglutination slide test (D-Di test, Diagnostica Stago S.A.S., France).

The other tube containing citrated whole blood was used for sonoclot analysis. 340 *μ*L of citrated whole blood was added to gb ACT+ (glass bead activated ACT) cuvette prewarmed to 37°C along with 20 *μ*L of CaCl_2_. Sonoclot signature was obtained and recorded for a period of 30 minutes on sonoclot analyzer (Sonoclot Coagulation and Platelet Function Analyzer, Sienco Inc., Arvada, CO, USA).

**Figure 2:**
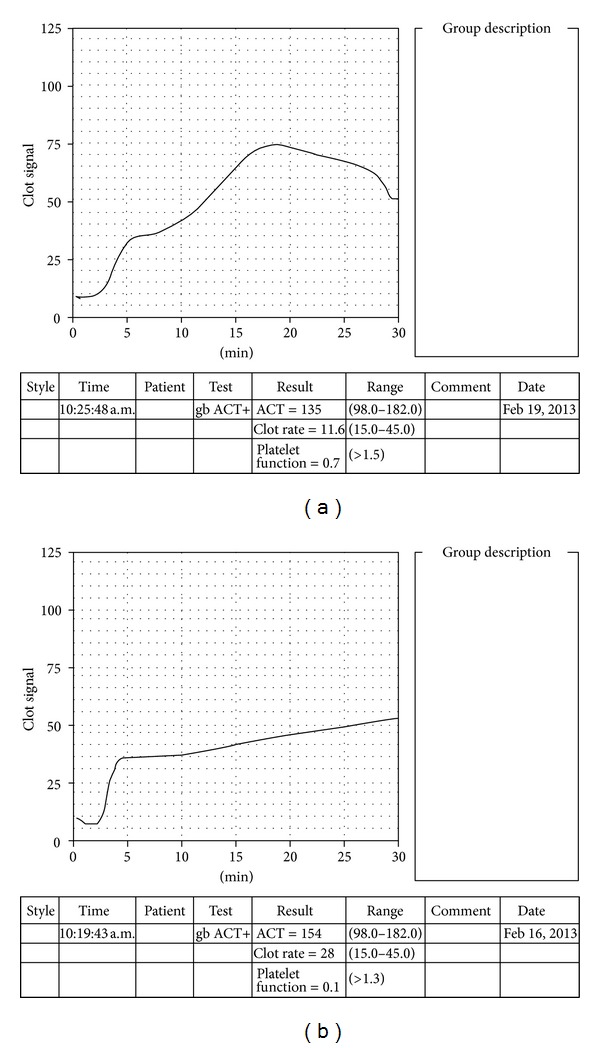
(a) Dull rounded peak on sonoclot signature. (b) Flat sonoclot signature.

SONACT, CR, and PF were calculated by the instrument and recorded accordingly. TP and PA were calculated manually from the signature. The R2 peak character was recorded as a qualitative parameter according to the type of peak obtained on the R2 slope of the signature. R2 peaks were classified as sharp (well-defined peaks, Figure 1), dull (poorly defined peaks, Figure 2(a)), and flat signature (Figure 2(b)).

### 3.4. Statistical Methods

The coagulation profiles of the patients as well as controls were groupwise tabulated. Quantitative data in different groups were expressed by median, mean, and standard deviation. Qualitative data was expressed as percentages. The sonoclot parameters obtained for different groups were compared with controls using the Wilcoxon signed rank test. The statistically significant difference between the patient groups and controls was reported using the Wilcoxon critical values table (at alpha = 0.05 level). Correlations between sonoclot parameters and conventional coagulation tests were calculated using Spearman's rank correlation and calculated *P* value. *P* value was considered significant if less than 0.05.

## 4. Results

Sonoclot signature parameters in different groups of liver disease as well as control group were studied.

*SONACT.* SONACT prolongation was seen maximally in G1 (D. cirrhosis) followed by the G4 (ACLF). The variations in SONACT values were also most pronounced in G1 (D. cirrhosis) as compared to other groups. A statistically significant difference was obtained between the SONACT values in patient groups as compared to controls (Table 2). 

*CR.* Mean value of CR was highest in G3 (cirrhosis) and lowest in G1 (D. cirrhosis). The control group was most consistent showing minimal variations in CR. The CR values in patient groups demonstrated a statistically significant difference as compared to the control group (Table 2). 

*PF.* Mean value of PF was lowest in G4 (ACLF) followed by G1 (D. cirrhosis). The difference in PF values between control group and G2 (CH) did not reach levels of statistical significance while the other groups depicted a statistically significant difference as compared to controls (Table 2). 

*TP.* Mean value of TP was the highest in G1 (D. cirrhosis) followed by G4 (ACLF). Deviations from the mean value were much less in the control group. Again, the difference in values between control group and G2 (CH) did not reach levels of statistical significance (Table 2). 

*PA.* Mean value of PA was lowest in G4 (ACLF) closely followed by G1 (D. cirrhosis). All the patient groups except for G2 (CH) demonstrated a statistically significant difference as compared to the control group (Table 2).

### 4.1. R2 Peak Character

**Figure 3:**
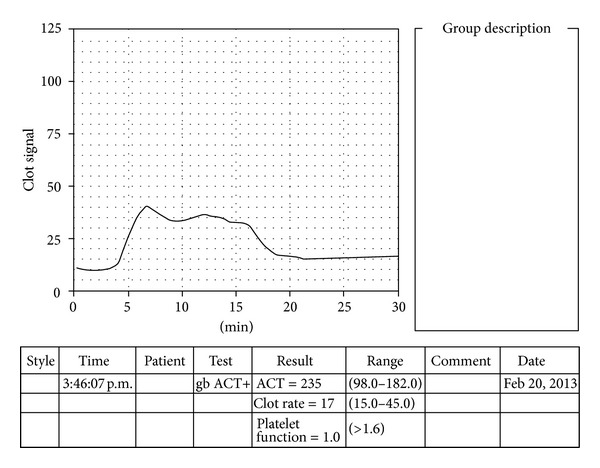
Hyperfibrinolysis as detected on sonoclot signature. The characteristic rise of the signature as depicted by R2 peak (suggestive of fibrin gel tightening by platelets) is not seen. Platelet function is subnormal (as calculated from the R3 gradient by the analyser). The hyperfibrinolysis in this case was confirmed by inspecting the sample in the cuvette immediately after the test procedure and was found to be in liquid state.

All the R2 peaks in the control group were sharp, well-defined peaks. In contrast, G1 (D. cirrhosis) and G4 (ACLF) showed grossly abnormal R2 peaks with G1 (D. cirrhosis) having around half of the patient population with dull peaks and maximum numbers of flat sonoclot signatures and G4 (ACLF) having around two-thirds of the patients with dull, poorly defined R2 peaks and few flat signatures as well (Table 3). One patient in G1 (D. cirrhosis) (NASH induced cirrhosis) depicted hyperfibrinolysis on sonoclot signature (Figure 3).

Sonoclot parameters and conventional coagulation tests were correlated and studied in patients with liver disease. A significant positive correlation was found between PT-INR, APTT and SONACT (*r* = 0.36, *P* < 0.008 (PT) and *r* = 0.43, *P* < 0.0015 (APTT)) and TP (*r* = 0.49, *P* < 0.0002 (PT) and *r* = 0.34, *P* < 0.01 (APTT)). PT and APTT were found to weakly correlate with CR and PA (*r* = −0.46, *P* < 0.0006 and *r* = −0.36, *P* < 0.008, resp.). As the coagulation was activated by glass beads in the sonoclot analyser, these parameters may not accurately correlate with PT and APTT. We also found a significant positive correlation between fibrinogen levels, platelet counts and CR, PF, PA and a significant negative correlation between these two conventional parameters and SONACT and TP. Regarding the D-dimer levels, statistically significant levels were not obtained with any of the sonoclot parameters (Table 4).

Similar correlations were also obtained between the coagulation variables (both sonoclot and conventional) and history of bleed without reaching levels of statistical significance.

## 5. Discussion

The coagulopathy of liver disease is complex and often unpredictable. While coagulopathy is the hallmark of ACLF group, the diagnostic tests of coagulation are frequently abnormal in patients with decompensated cirrhosis too [11]. Most of the sonoclot parameters in our study also demonstrate statistically significant abnormalities in decompensated cirrhotics and ACLF group while the noncirrhotic category shows comparatively fewer abnormalities. Prolonged SONACT and shortened CR in these cases can be attributed to decreased synthesis of Vitamin K dependent factors (II, VII, IX, and X). Additionally, prolonged TP and decreased PF could be a result of thrombocytopenia caused by splenic sequestration. Also, impaired platelet aggregation responses to adenosine diphosphate, arachidonic acid, collagen, and thrombin lead to low PF values on sonoclot analysis in chronic liver disease [12].

Despite clear evidence of an increased tendency for bleeding in patients with liver disease, many circumstances also promote local and systemic hypercoagulable states [11, 13].

The routine coagulation tests give no insight regarding the hypercoagulable tendency in patients with liver disease. Due to lack of proper measurement tools to identify those patients who are prone to develop clots, there is reliance on clinical endpoints like deep vein thrombosis, portal vein thrombosis, and so forth to detect the presence of hypercoagulability in these patients [14].

**Figure 4:**
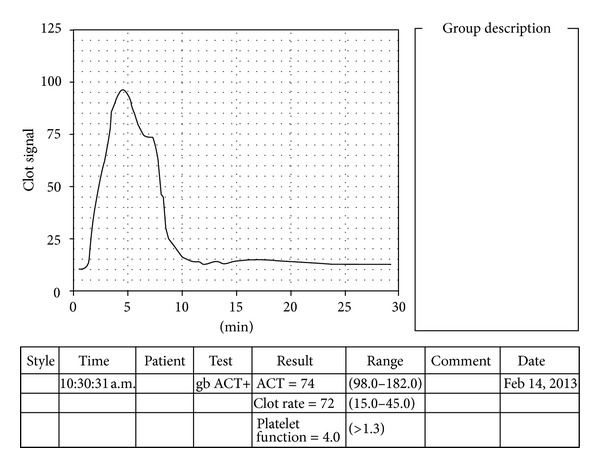
Hypercoagulability (ACT of sample < ACT normal range, CR of sample ≫ CR normal range). The steep slope of the signature is depicted by increased clot rate (72 units/min). The characteristic rise of the graph (as seen by increased peak amplitude) and sharp peak depict strong clot retraction by platelets. Note also the time to peak which is very short (<5 minutes). The platelet function is very good (as calculated by the R3 gradient).

Assessment of prothrombotic states has been carried out in cancer patients, using sonoclot analyser, by studying significantly increased CRs. This study used powdered celite as contact activator instead of glass beads [15]. In our study, the highest mean CR was observed in the cirrhotic HCC group followed by the chronic hepatitis group (Table 2). Some of the CRs of the patients in these groups were quite high as compared to the control group (reaching as high as 72 units/min in one of the patients (Figure 4) (biological reference range: 15–45 units/min)). These abnormal values could help to define the underlying state of hypercoagulability in these patients. As our study did not include the subsequent followup of these cases, the predictive value of CR regarding the prothrombotic tendency cannot be demonstrated with certainty in these patients. Nevertheless, this sonoclot parameter has ample potential to be explored as a predictor of hypercoagulable state in liver diseases.

Hypercoagulable states in cirrhosis have been attributed to decrease in the levels of natural anticoagulant proteins (protein C, protein S, and antithrombin III) and increase in factor VIII and von Willebrand factor levels [1]. Additionally, it is suggested that prothrombotic tendencies are common in HCC patients due to the ability of tumor cells to secrete procoagulants/fibrinolytic inhibitor factors and inflammatory cytokines [16]. Also, elevated homocysteine levels in patients with HCC have been implicated in thromboembolic tendencies [16]. (Whether the coagulation abnormalities detected in cirrhotic-HCC group in our study were purely because of cirrhosis or influenced by an additional finding of HCC or due to both could not be assessed as the number of cases in this group was not sufficient for such analysis).

The correlation between conventional coagulation tests and sonoclot parameters is rather limited [17, 18]. In this study we have tried to compare the sonoclot parameters with conventional tests so as to be able to replace the need for several coagulation tests in these patients with sonoclot. SONACT and TP have shown a statistically significant positive correlation with PT-INR and APTT and a statistically significant negative correlation with platelet count and fibrinogen levels, whereas CR and PA have demonstrated a statistically significant negative correlation with PT-INR and APTT and a highly significant positive correlation with fibrinogen levels and platelet count. These findings are in agreement with other studies aiming to establish similar correlations [19, 20]. These sonoclot variables once obtained, may subsequently be used to predict the PT-INR and APTT values as well as fibrinogen levels in liver disease patients.

The D-dimer levels in this study have not shown any statistically significant correlation with the sonoclot parameters. D-dimer levels may act as marker for enhanced fibrinolytic activity and disseminated intravascular coagulation (DIC) [21]. However, the increased levels do not always indicate hyperfibrinolysis. In our study, eight patients had very high levels of plasma D-dimers out of which only one patient had hyperfibrinolytic tracing on sonoclot signature. This finding may be related to some studies which show that, in spite of increased levels of D-dimer, actual incidence of hyperfibrinolysis in patients of cirrhosis is quite less and elevated levels of D-dimers may merely be because of coagulation activation cascade [22, 23]. Certain studies have pointed out that the indicators of fibrinolysis such as D-dimer, are breakdown products which have a relatively short half-life. It is likely that the clearance of these molecules is delayed in patients of liver disease resulting in falsely elevated D-dimer levels [24].

As sonoclot effectively measures global hemostasis by monitoring the viscosity changes in blood during initiation of coagulation and development of clot [25], it is immune to the levels of by-products of fibrin breakdown in plasma and may prove to be a better method for detection of hyperfibrinolysis.

Hyperfibrinolysis can be accurately assessed by TEG [26, 27]. As both TEG and sonoclot are based on similar principles, fibrinolysis as detected by sonoclot may be comparable to TEG fibrinolysis. However, it is important to note that fibrinolysis might not always be apparent on sonoclot and localized clot breakdown by plasmin may not be depicted by this method. Hence, the sensitivity of sonoclot to detect the process of fibrinolysis needs to be studied further especially in conjunction with other markers of fibrinolysis before substantiating the importance of this test in cases of hyperfibrinolysis.

As it has already been described in several studies [28, 29], none of the standard tests of coagulation in this study depicted any significant correlation with history of bleed in patients with liver disease. Unfortunately, none of the sonoclot parameters also had any statistically significant correlation with bleeding history in these patients.

Alternative tests such as thromboelastography (TEG), thrombin generation test (TGT), and clotting factor assays should be explored to predict bleeding and hypercoagulability in liver disease. TEG has been applied in liver disease patients to assess global coagulation. Stravitz et al. have noted normal TEG parameters in spite of prolonged PT-INR values in liver injury patients [30].

TEG has certain disadvantages like increased failure rates of the test procedure. In our experience, sonoclot has proved to be more durable than TEG requiring fewer repetitions. Also, SONACT is considered more specific as it represents the initial clot formation and reflects clotting factor defects whereas TEG reaction time (R) gives information about a more mature and developed fibrin clot [31].

Individual factor assays have also been used to define hemostatic disorders in liver disease but their main disadvantage is that they do not give information on the entire coagulation process. Also, they are more time-consuming and expensive and may show significant interlaboratory variations [32]. In contrast, sonoclot provides overall picture of coagulation profile in a single test procedure.

Hemostatic defects in liver disease have been assessed by several coagulation measures including individual factor assays, TEG and sonoclot. Not only is the assessment of factor levels cumbersome, but it also fails to give complete information on the coagulation system. TEG, like sonoclot, is a global assay of coagulation but the reaction time obtained does not accurately define the initial clotting process. Hence, sonoclot assay is quite an accurate tool, comparable to TEG for assessing global coagulopathic disorders in liver disease.

TGT has also shown utility in assessing bleeding or thrombotic risks and may be used to predict the coagulopathy of liver disease [33]. Studies have shown that the balance of procoagulants and anticoagulants in patients with cirrhosis was normal when thrombin generation was measured in presence of thrombomodulin even though PT and APTT were prolonged [34]. Hence, this test should be developed in appropriate clinical trials as a predictor of bleeding as well as thrombosis in liver disease [35].

## 6. Conclusion

Sonoclot analyzer can be used as an effective tool to assess coagulation defects in liver disease patients as statistically significant differences are obtained on sonoclot signatures especially in the ACLF and decompensated cirrhotic groups as compared to normal healthy individuals.

The statistically significant correlations between routine tests and sonoclot parameters prove that this global test of coagulation should be used in conjunction with the standard tests to define the hemostatic profile in liver disease as conventional tests alone have shown poor reproducibility with bleeding and thrombotic risks. CR on sonoclot signature may prove to be an effective guide for predicting thrombosis in patients of liver disease in the future. Similarly, the detection of hyperfibrinolysis on sonoclot needs to be explored further in relation with TEG and plasma D-dimer levels.

## Figures and Tables

**Table 1:** Demographic and clinical data.

Demographic data	Patient groups (*N* = 50)		Control group (*N* = 10)	
Male : female	34 : 16		6 : 4	
Age mean ± SD	50.7 ± 10.68		34.2 ± 9.4	

Clinical data	Patient groups			
	G1 (*N* = 16)	G2 (*N* = 14)	G3 (*N* = 11)	G4 (*N* = 09)

History of bleed	4 (25%)	1 (7%)	1 (9%)	—
Hepatic encephalopathy	8 (50%)	3 (21%)	—	4 (44%)
Sepsis	6 (37.5%)	3 (21%)	1 (9%)	3 (33%)

G1: group 1 (decompensated cirrhosis), G2: group 2 (noncirrhotic liver disease), G3: group 3 (cirrhotic HCC), G4: group 4 (ACLF).

**Table 2:** Comparison between sonoclot parameters in different groups.

Group		G1	G2	G3	G4	Control
SONACT	Mean ± SD (s)	176.31 ± 51.41	146.14 ± 41.76	130.09 ± 31.23	152.44 ± 13.22	137 ± 24.46
	Median (s)	164	147	140	148	143.5
	*W*_*C*_	88*	27*	22*	23*	—

CR	Mean ± SD (u/min)	29.41 ± 11.31	42.5 ± 17.64	44.54 ± 10.44	34.88 ± 11.54	31.6 ± 7.63
	Median (u/min)	30	44.5	48	30	44
	*W*_*C*_	50*	59*	56*	11*	—

PF	Mean ± SD	1.54 ± 1.08	2.29 ± 1.31	2.97 ± 0.84	1.36 ± 1.13	2.53 ± 0.76
	Median	1.5	2.7	3.0	1.4	2.45
	*W*_*C*_	98*	07^¶^	45*	31*	—

TP	Mean ± SD (min)	20.5 ± 10.2	11.5 ± 7.6	9.5 ± 10.4	15.3 ± 11.9	11.8 ± 2.8
	Median	17	9.5	6	10	11.5
	*W*_*C*_	116*	18^¶^	44*	11*	—

PA	Mean ± SD (units)	75.31 ± 25.13	91.07 ± 21.94	97.91 ± 8.61	71.94 ± 24.99	95.7 ± 7.48
	Median	80	95	100	75	93.5
	*W*_*C*_	88*	05^¶^	32*	33*	—

	*W*_*T*_	29	21	10	05	—

G1: group 1 (decompensated cirrhosis), G2: group 2 (noncirrhotic liver disease), G3: group 3 (cirrhotic HCC), G4: group 4 (ACLF).

SONACT: sonoclot activated clotting time, CR: clot rate, PF: platelet function, TP: time to peak, PA: peak amplitude.

SD: standard deviation.

*W*_*C*_: calculated Wilcoxon test statistic (patient groups versus controls), *W*_*T*_: tabulated Wilcoxon critical value of alpha = 0.05 (*W*_*c*_ > *W*_*T*_ is considered to be statistically significant).

*Significantly different values as compared to control group.

^¶^Values are not significantly different as compared to controls.

**Table 3:** Comparison between R2 peak characters in different groups.

R2 peak in	G1	G2	G3	G4	Control group
Sharp peak (%)	18.75	71.43	90.9	11.11	100
Dull peak (%)	50	7.14	9.1	66.67	00
Flat (%)	31.25	21.43	0	22.22	00

G1: group 1 (decompensated cirrhosis), G2: group 2 (noncirrhotic liver disease), G3: group 3 (cirrhotic HCC), G4: group 4 (ACLF).

**Table 4:** Correlation obtained between sonoclot parameters and conventional coagulation variables in patients with liver disease.

Conventional tests		PT-INR	APTT	Fibrinogen	D-dimer	Platelet count
Sonoclot parameters	*P* value (*r*: correlation coefficient)					
	SonACT	<0.008 (*r* = 0.36)	<0.0015 (*r* = 0.43)	<0.037 (*r* = −0.29)	0.39 (*r* = 0.12)	<0.01 (*r* = −0.34)
	CR	<0.0025 (*r* = −0.41)	<0.0025 (*r* = −0.41)	<0.004 (*r* = 0.39)	0.96 (*r* = −0.005)	<0.03 (*r* = 0.3)
	PF	0.07 (*r* = −0.25)	0.2 (*r* = −0.16)	<0.001 (*r* = 0.44)	0.89 (*r* = 0.02)	<0.0001 (*r* = 0.62)
	TP	<0.0002 (*r* = 0.49)	<0.01 (*r* = 0.34)	<0.0001 (*r* = −0.56)	0.12 (*r* = 0.22)	<0.0001 (*r* = −0.61)
	PA	<0.0006 (*r* = −0.46)	<0.008 (*r* = −0.36)	<0.0001 (*r* = 0.56)	0.6 (*r* = −0.08)	<0.0002 (*r* = 0.5)

PT: prothrombin time, INR: international normalized ratio, APTT: activated partial thromboplastin time.

SONACT: sonoclot activated clotting time, CR: clot rate, PF: platelet function, TP: time to peak, PA: peak amplitude.

*r*: Spearmans rank correlation coefficient.

*P* value is significant if <0.05.
